# Immunological Tolerance after Living-Donor Liver Transplantation from the Same Donor as for a Prior Bone Marrow Transplant: A Case Report without Rejection or Graft-Versus-Host Disease

**DOI:** 10.70352/scrj.cr.26-0503

**Published:** 2026-11-05

**Authors:** Tomokazu Tokoro, Satoshi Takada, Kaoru Katano, Hiroaki Sugita, Ryosuke Gabata, Ryohei Takei, Mitsuyoshi Okazaki, Akihiro Seki, Shinichi Nakanuma, Isamu Makino, Kouichiro Uchida, Hirohito Yamazaki, Shintaro Yagi

**Affiliations:** 1 Department of Hepato-Biliary-Pancreatic Surgery and Transplantation/Pediatric Surgery, Kanazawa University, Kanazawa, Ishikawa, Japan; 2 Department of Gastroenterology, Kanazawa University, Kanazawa, Ishikawa, Japan; 3 Juntendo Advanced Research Institute for Health Science, Juntendo University School of Medicine, Tokyo, Japan; 4 Department of Hematology, Kanazawa University, Kanazawa, Ishikawa, Japan

**Keywords:** LDLT, GVHD, Immunological tolerance

## Abstract

**INTRODUCTION:**

We report a unique case of successful early weaning off immunosuppression following living-donor liver transplantation from the same donor as for a prior bone marrow transplantation, without the development of rejection or graft-versus-host disease.

**CASE PRESENTATION:**

The patient, a 45-year-old man, underwent bone marrow transplantation at ages 12 and 14 for myelodysplastic syndrome, receiving hematopoietic stem cells from his human leukocyte antigen-identical older brother. Following the second bone marrow transplantation, the patient did not experience myelodysplastic syndrome recurrence and successfully discontinued immunosuppressive therapy. Decades later, he developed hepatitis C cirrhosis complicated by multiple hepatocellular carcinomas, necessitating living-donor liver transplantation. His brother, the original bone marrow transplantation donor, served as the liver donor; they shared an identical blood type and a full human leukocyte antigen match. Post–living-donor liver transplantation, the patient received methylprednisolone for 1 week only, after which all immunosuppression was ceased. At 30 months post-transplant, the patient remains free of rejection, graft-versus-host disease, and hepatocellular carcinoma recurrence, suggesting the establishment of long-term immunological tolerance.

**CONCLUSIONS:**

This case highlights the potential for immunosuppression-free liver transplantation when the donor and recipient share a full human leukocyte antigen match and prior bone marrow engraftment, offering important insights for the field of transplant immunology.

## Abbreviations

AFP
: alpha-fetoprotein
BMT
: bone marrow transplantation
CNIs
: calcineurin inhibitors
EOB-MRI
: ethoxybenzyl-enhanced MRI
GVHD
: graft-versus-host disease
HLA
: human leukocyte antigen
LDLT
: living-donor liver transplantation
MDS
: myelodysplastic syndrome
MLR
: mixed lymphocyte reaction
TACE
: transcatheter arterial chemo-embolization

## INTRODUCTION

The first successful kidney transplant in the world was performed between 2 identical twins, and the recipient reported no rejection without the use of immunosuppressive drugs.^1)^ Since then, there have been many reports of successful organ transplants without the use of immunosuppressive drugs.^2)^ We experienced a case in which a patient underwent BMT in childhood and, as an adult, underwent LDLT from the same donor. The donor’s bone marrow was fully viable, and, theoretically, rejection or GVHD would not be expected to occur; however, a case like this has not previously been reported, so the real-world clinical outcome was unknown. In particular, there have been no reports of how donor lymphocytes, which engrafted in a recipient after BMT during childhood and were educated in the recipient thymus, affect a transplanted liver approximately 30 years later, or how passenger T cells in the transplanted liver behave in such a recipient’s body. We believe that the results of this case are extremely valuable and that this report will contribute to the development of liver transplantation medicine.

## CASE PRESENTATION

The patient underwent BMT for MDS at the ages of 12 and 14, with his older brother as the donor. Since then, there has been no MDS relapse, and immunosuppression was completed. At the age of 39, the patient developed hepatocellular carcinoma (HCC) in hepatitis C viral cirrhosis, for which he underwent left hepatectomy and S8 partial hepatectomy. After that, he underwent transcatheter arterial chemoembolization 4 times for HCC recurrence. At the age of 45, owing to liver cirrhosis and HCC (maximum diameter: 2.3 cm; tumor number: 3), he underwent LDLT from the same donor with a right liver graft. Preoperative evaluation was based on contrast-enhanced CT performed within 1 month prior to LDLT. Preoperative contrast-enhanced CT revealed 3 nodules showing typical HCC features, including intense enhancement and washout, including a local recurrence following TACE (**Fig. 1**). These imaging findings were reviewed preoperatively during a multidisciplinary conference involving a radiologist, and LDLT was performed after determining that the HCC met the Japanese criteria. The donor blood type was identical, and lymphocyte crossmatch tests were negative by both flow cytometry lymphocyte crossmatch and lymphocyte cytotoxicity test crossmatch. HLA tests showed a full match between the donor and recipient (**Table 1**), and there was low activity in a MLR (**Fig. 2**). The standard liver volume (SLV) was 1224 mL, the right lobe graft volume (GV) was estimated as 962 mL, the graft-to-recipient body weight ratio (GRWR) was 1.27%, the GV/SLV ratio was 78.6%, and the donor residual liver rate was 32.2%. Prior to implantation, the V5 and the right hepatic vein root were reconstructed using an inferior mesenteric vein harvested from the donor on the back table. The transplant procedure began with hepatic vein anastomosis. This was followed by portal vein anastomosis. As there was insufficient distance for anastomosis between the portal vein stumps of the graft and the recipient, the decision was made to place an interposed graft. The recipient’s left external iliac vein was harvested to create an interposed graft, and an anastomosis was performed between the graft and the vessel. Blood flow to the grafted liver was then restored. For the hepatic artery anastomosis, the recipient’s right hepatic artery was anastomosed to the graft’s right hepatic artery. Bile duct anastomosis was performed using an end-to-end anastomosis. The operative time was 19 hours and 9 minutes, blood loss was 2040 g, the cold ischemia time was 362 minutes, and the warm ischemia time was 34 minutes. Methylprednisolone was administered as a perioperative immunosuppressive treatment at 500 mg/body for intraoperative administration, 1 mg/kg on PODs 1 to 3, and 0.5 mg/kg on days 4 to 6. A liver graft biopsy was performed on POD 5, which revealed no T cell-mediated rejection or antibody-mediated rejection (**Fig. 3**). The patient was discharged 50 days after surgery. Pathological diagnosis for HCC showed multiple HCC tumors (maximum diameter: 2.5 cm; tumor number: 12) and, separately, a single cholangiocarcinoma (diameter: 0.2 cm) (**Fig. 1**). The HCC consisted primarily of well- to moderately differentiated cells (**Fig. 4A** and **4B**). Peripheral hepatic vein invasion (vv1) was observed in the large tumor, but no vascular invasion was detected in the other HCCs. A multicentric origin is suspected. The 0.2-cm lesion consisted of proliferating epithelium resembling small bile ducts arranged in tubular and cord-like patterns (**Fig. 4C**). It did not contain HCC components. Immunohistochemical staining showed negative results for hepatocyte and arginase-1, and positive results for CK7 (**Fig. 4D**), CK19, and CD56 (**Fig. 4E**). EMA (**Fig. 4F**) staining was positive in some areas, but findings of positivity on the luminal side of small lumens were limited to only a very small portion; hyaline stroma was also scanty. Based on the above findings, the diagnosis was small duct-type intrahepatic cholangiocarcinoma. The patient’s post-discharge course was uneventful. We performed liver biopsies at 7 and 14 months after the surgery and found no evidence of rejection (rejection activity index (RAI) score: 0) or GVHD. At present, 30 months after the surgery, the patient shows no recurrence of HCC without immunosuppression.

**Fig. 1:**
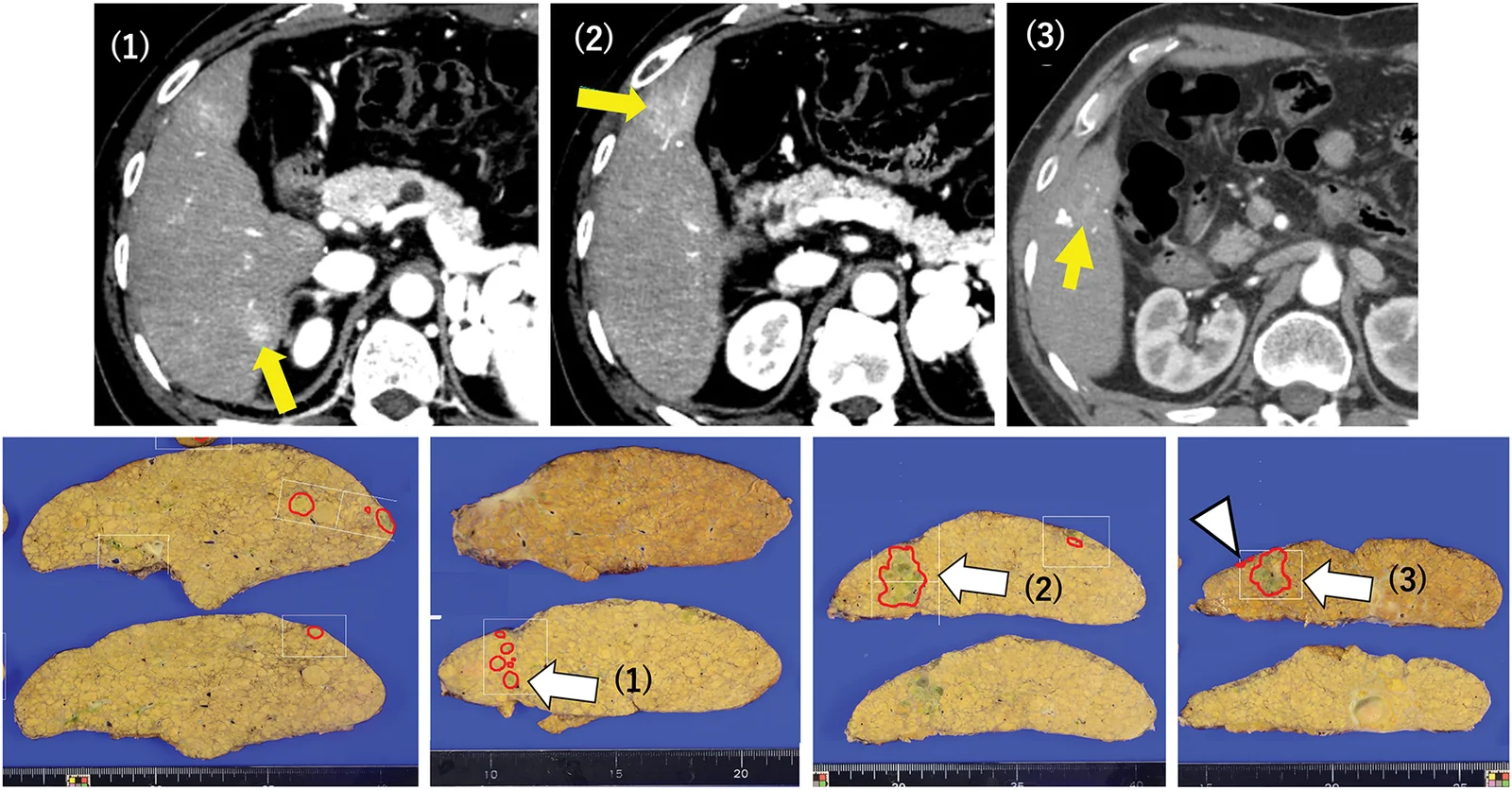
CT image findings and photographs of the resected specimen. Three HCC lesions (**1**–**3**) were identified on contrast-enhanced CT (yellow arrows). Examination of the resected specimen revealed numerous additional HCC lesions (red enclosed area) and one small intrahepatic cholangiocarcinoma lesion of the bile duct type (white arrowhead), in addition to the 3 HCC lesions noted preoperatively (white arrows). HCC, hepatocellular carcinoma

**Fig. 2:**
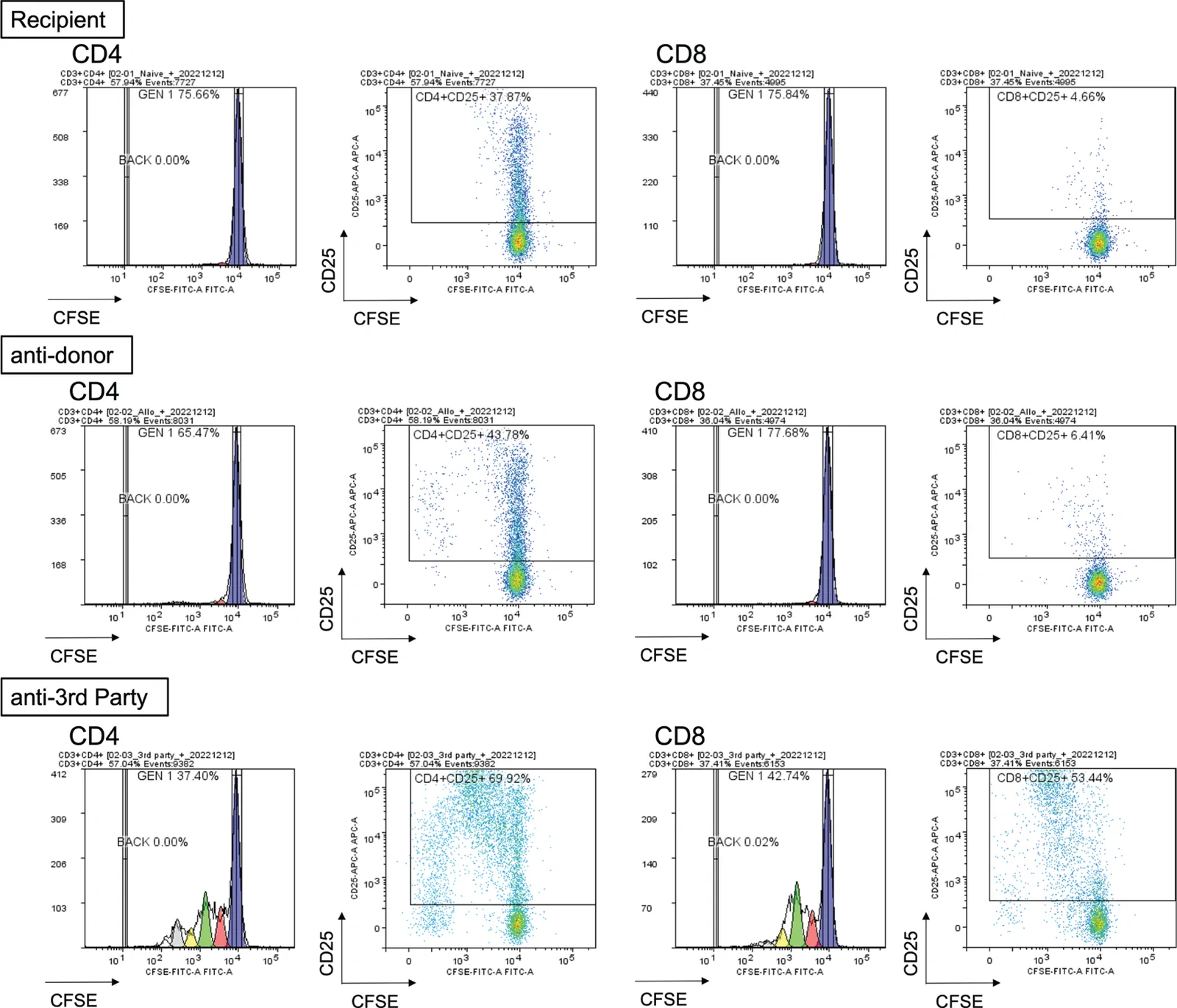
Mixed leukocyte reaction. Evaluation of the anti-donor immune response by CFSE-mixed leukocyte reaction. Flow cytometry profiles after CFSE-mixed leukocyte reaction using donor and third-party stimulators, showing the expression of CD25 by CD4+ T cells and CD8+ T cells. Unstimulated recipient cells served as the negative control. The response to donor lymphocytes was extremely low. CFSE, carboxyfluorescein succinimidyl ester

**Fig. 3:**
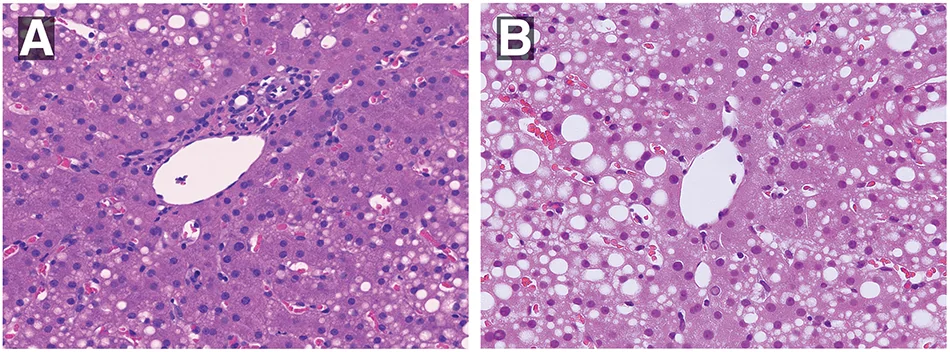
Histopathological examination of a liver biopsy. No, inflammation of the portal vein area (Zone 1), bile duct abnormalities (**A**), or venous endotheliitis was observed in the pericentral area (Zone 3) (**B**), and the RAI score was assessed as 0 (HE staining: ×200). HE, hematoxylin and eosin stain; RAI, rejection activity index

**Fig. 4:**
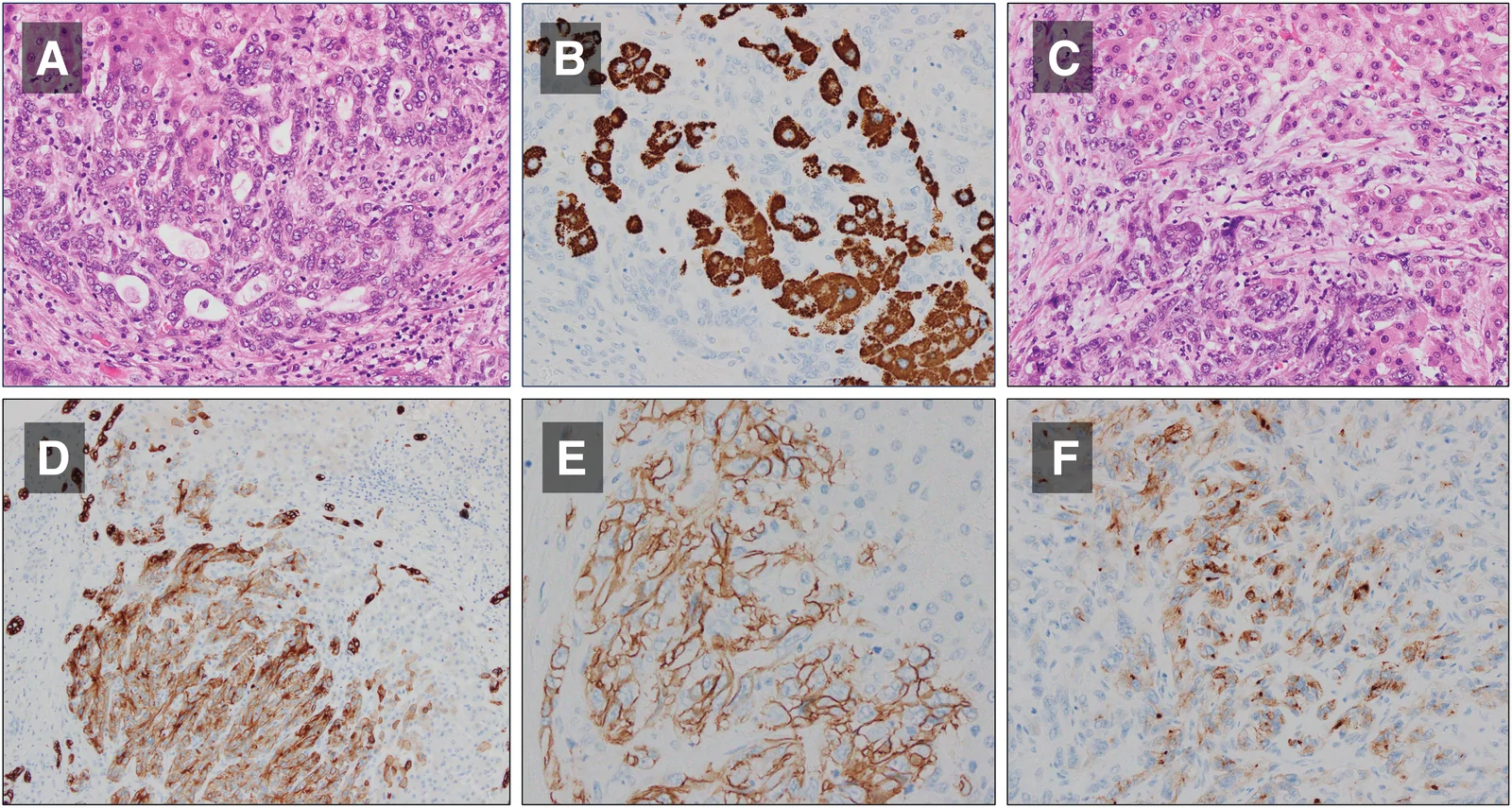
Immunohistochemical examination. The tumor was a well-differentiated HCC (HE staining: ×200; **A**), and immunohistochemical analysis showed positivity for hepatocytes (×200; **B**). The 0.2-cm lesion consisted of proliferating epithelium resembling small bile ducts arranged in tubular and cord-like patterns (HE staining: ×200; **C**), and immunohistochemical analysis showed positivity for CK7 (×200; **D**), CD56 (×200; **E**), and EMA (×200; **F**). HCC, hepatocellular carcinoma; HE, hematoxylin and eosin stain

**Table 1:** Results of HLA testing

HLA antigen	HLA-A		HLA-B		HLA-C	
Recipient	02:01:01	26:03:01	15:01:01	40:01:02	03:04:01	–
Donor	02:01:01	26:03:01	15:01:01	40:01:02	03:04:01	–
	HLA-DRB1		HLA-DRB3/4/5		HLA-DQA1	
	04:05:01	11:01:01	3*02:02:01	4*01:03:01	03:03:01	05:05:01
	04:05:01	11:01:01	3*02:02:01	4*01:03:01	03:03:01	05:05:01
	HLA-DQB1		HLA-DPA1		HLA-DPB1	
	03:02:01	04:01:01	01:03:01	–	02:01:02	03:01:01
	03:02:01	04:01:01	01:03:01	–	02:01:02	03:01:01
	HLA-A		HLA-B		Bw	
	A2	A26	B62	B60	6	–
	A2	A26	B62	B60	6	–
	HLA-C		HLA-DR		HLA-DRB3/4/5	
	Cw10	–	DR4	DR11	DR52	DR53
	Cw10	–	DR4	DR11	DR52	DR53
	HLA-DQ		Haplo			
	DQ8	DQ4	a/c			
	DQ8	DQ4	a/c			

Both the antigen type and the genotype were a full match.

HLA, human leukocyte antigen

## DISCUSSION

BMT, a treatment for hematologic malignancies and immunodeficiency, results in the complete replacement of the recipient’s hematopoietic system with that of the donor.^3)^ Therefore, if an organ transplant is also performed from the same donor after BMT, the donor-replaced hematopoietic system should be immunologically tolerant to the transplanted organ in the recipient’s body, and rejection is not expected to occur. However, there have been no reports of living partial liver transplantation from the same donor after BMT. In this case, the patient received BMT as a child and underwent LDLT from the same donor as an adult. HLA tests showed that both the antigen type and genotype were fully matched, as expected. The MLR is a test to evaluate the response of T lymphocytes via a direct recognition pathway to the same type of major histocompatibility complex.^4)^ In this case, the response to donor lymphocytes was extremely low. Looking at the results in detail, compared with its expression in unstimulated cells, CD25 was expressed during donor stimulation, with a low level of CD4 and the appearance of mild proliferation. The CD8 expression was virtually absent. With reference to the report by Tanaka et al.,^4)^ this case is considered to have such low reactivity that the initial dose of immunosuppressive drugs could be reduced. Although there was very mild donor responsiveness, we considered the risk of rejection in this case to be extremely low. These test results suggest that immunological tolerance to the donor has been established in the recipient’s body.

Although the mechanism of immunological tolerance is not yet clear, negative selection in the thymus, regulatory T cell activity, and suppressive cytokines have been suggested.^5,6)^ Furthermore, while the recipient in this case had a unique immunological background that could result in donor-derived immune reconstitution and donor-specific tolerance, donor-derived naïve T cells contained within the liver graft may theoretically contribute to GVHD. In addition, if the recipient’s body maintained immune tolerance through regulatory T cells or suppressive cytokines, liver transplantation could disrupt this balance, potentially triggering both rejection and GVHD.

On the other hand, it is said that if donor-derived B lymphocytes contained in a transplanted liver remain in the recipient’s body, the recipient may develop passenger lymphocyte syndrome (PLS). PLS is primarily caused by donor-derived B lymphocytes that produce antibodies against the recipient’s red blood cell antigens, and it is an immunological phenomenon distinct from GVHD.^7)^ In this case, the patient underwent BMT during childhood. Since it is believed that, following a hematopoietic stem cell transplant, the patient’s red blood cells are ultimately replaced by those from the donor,^8)^ PLS is considered unlikely to develop if red blood cell replacement has been achieved.

While there are indications that immunosuppressants may be discontinued in cases where chimerism is achieved, this is still in the research stage.^9)^ There is no established protocol for immunosuppressive therapy in extremely rare cases such as this one. We adopted a policy of using steroids alone during the perioperative period and carefully monitoring the patient postoperatively—including liver biopsies—for signs of rejection; if any signs of rejection were observed, we would administer CNIs. After discontinuing steroid therapy, we confirmed through clinical examination, blood tests, and liver biopsies that there were no signs of rejection or GVHD, and we were thus able to achieve immunosuppressant-free management.

In this case, preoperative imaging evaluation had diagnosed 3 HCCs, but the pathological findings from the resected specimen revealed 12 HCCs as well as a 0.2-cm intrahepatic cholangiocarcinoma, indicating a significant discrepancy between the 2 assessments. Even upon reviewing the preoperative CT scans, preoperative imaging diagnosis of the multiple small lesions proved extremely difficult in this case, given the underlying liver cirrhosis characterized by regenerative nodules. It has been reported that EOB-MRI demonstrates higher sensitivity than contrast-enhanced CT, particularly in the detection of small HCCs.^10)^ In this case, a preoperative EOB-MRI evaluation was not performed; we believe that had EOB-MRI been added, it might have detected some of the small nodules that were not identified on contrast-enhanced CT.

A nationwide survey in Japan reported a 5-year HCC recurrence rate of 9.1% among cases meeting either the Milan criteria or the 5-5-500 rule within the Japanese criteria.^11)^ However, postoperative pathological findings in this case showed that the patient met neither the Milan criteria nor the 5-5-500 criteria. Mehta et al.^12)^ have proposed a scoring system to evaluate the risk of post-transplant recurrence based on AFP levels, the presence or absence of microvascular invasion in the explanted liver, and tumor size; according to this system, the patient falls into the high-risk group for recurrence. Although the patient remains recurrence-free 30 months post-surgery, careful follow-up is considered necessary.

## CONCLUSIONS

We successfully performed LDLT decades after BMT from the same donor, with very early weaning off immunosuppression without rejection or GVHD.

## DECLARATIONS

### Availability of data and materials

The data that support the findings of this study are available from the corresponding author upon reasonable request.
